# Linkage analysis of obesity phenotypes in pre- and post-menopausal women from a United States mid-western population

**DOI:** 10.1186/1471-2350-11-156

**Published:** 2010-11-09

**Authors:** Linda E Kelemen, Elizabeth J Atkinson, Mariza de Andrade, V Shane Pankratz, Julie M Cunningham, Alice Wang, Christopher A Hilker, Fergus J Couch, Thomas A Sellers, Celine M Vachon

**Affiliations:** 1 Department of Population Health Research, Alberta Health Services-Cancer Care, Calgary, AB, Canada; 2 Department of Health Sciences Research, Mayo Clinic College of Medicine, Rochester, MN, USA; 3 Department of Laboratory Medicine and Pathology, Mayo Clinic College of Medicine, Rochester, MN, USA; 4 Department of Cancer Epidemiology and Genetics, H. Lee Moffitt Cancer Center and Research Institute, Tampa, FL, USA

## Abstract

**Background:**

Obesity has a strong genetic influence, with some variants showing stronger associations among women than men. Women are also more likely to distribute weight in the abdomen following menopause. We investigated whether genetic loci link with obesity-related phenotypes differently by menopausal status.

**Methods:**

We performed univariate and bivariate linkage analysis for the phenotypes of body mass index (BMI), waist (W) and hip (H) circumferences (WC, HC), and WH ratio (WHR) separately among 172 pre-menopausal and 405 post-menopausal women from 90 multigenerational families using a genome scan with 403 microsatellite markers. Bivariate analysis used pair-wise combinations of obesity phenotypes to detect linkage at loci with pleiotropic effects for genetically correlated traits. BMI was adjusted in models of WC, HC and WHR.

**Results:**

Pre-menopausal women, compared to post-menopausal women, had higher heritability for BMI (*h*^2 ^= 94% versus *h*^2 ^= 39%, respectively) and for HC (*h*^2 ^= 99% versus *h*^2 ^= 43%, respectively), and lower heritability for WC (*h*^2 ^= 29% versus *h*^2 ^= 61%, respectively) and for WHR (*h*^2 ^= 39% versus *h*^2 ^= 57%, respectively). Among pre-menopausal women, the strongest evidence for linkage was for the combination of BMI and HC traits at 3p26 (bivariate LOD = 3.65) and at 13q13-q14 (bivariate LOD = 3.59). Among post-menopausal women, the highest level of evidence for genetic linkage was for HC at 4p15.3 (univariate LOD = 2.70) and 14q13 (univariate LOD = 2.51). WC was not clearly linked to any locus.

**Conclusions:**

These results support a genetic basis for fat deposition that differs by menopausal status, and suggest that the same loci encode genes that influence general obesity (BMI) and HC, specifically, among pre-menopausal women. However, lower heritability among pre-menopausal women for WC and WHR suggests that pre-menopausal waist girth may be influenced to a greater extent by controllable environmental factors than post-menopausal waist girth. Possibly, targeted interventions for weight control among pre-menopausal women may prevent or attenuate post-menopausal abdominal weight deposition.

## Background

Over the past 25 years, the prevalence of overweight adults increased by 40% and that of obese adults by almost 2-fold in the United States [1]. Together, overweight and obese adults account for 68% of Americans [2]. Global increases in weight gain have also been reported [3], and mirror the rise in obesity-related chronic diseases including type 2 diabetes, cardiovascular diseases [4], and several cancers [5].

The rapid increases in overweight and obesity are multi-factorial and only partly attributable to changes in lifestyle practices [4]. Results from transgenic and crossbred animal models and population-based studies (reviewed in [6]), and more recently genome-wide association studies (GWAS) [7-14], provide strong evidence for genetic influences on obesity-related phenotypes, including overall weight gain and body fat distribution. Although family and twin studies estimate that 40-70% of the population variation in body mass index (BMI), a measure of overall adiposity, is due to genetic factors [15,16], genetic variants identified from the GWAS account for < 1% of the variance of BMI in those subjects [12,13], leaving a large proportion of the genetic variation unexplained.

Body fat distribution may be more important than overall adiposity on health [17]. Visceral abdominal fat composed of omental and mesenteric adipocytes is more metabolically active than subcutaneous fat [18], secreting a variety of cytokines and inflammatory agents with immunological, vascular, and metabolic actions [19]. Previous studies showed that health risks are positively associated with waist circumference but inversely related to hip circumference after adjustment for potential confounders [20,21]. Body composition changes occur frequently among women following menopause, in whom age-related increases in obesity occur more often [22]. Observations based on objective assessments of body composition such as CT scans noted increased visceral fat deposition in post-menopausal compared to pre-menopausal women [23]. Changes in body fat distribution during the menopausal transition have also been demonstrated longitudinally [24,25], including an absolute cumulative 6-year increase of approximately 5.7 cm in waist circumference [24]. Estrogen treatment has been shown to prevent the increase in intra-abdominal fat [26,27]. Possibly, the genetic determinants of abdominal fat deposition and its metabolic sequelae may differ from those that determine hip deposition of fat.

To our knowledge, no studies have examined genetic determinants of obesity among pre-menopausal and post-menopausal women separately. Because these groups appear to have different propensities for fat deposition, we used linkage analysis to test the hypothesis that obesity-related phenotypes are influenced by different genetic loci among pre-menopausal and post-menopausal women using data from the Minnesota Breast Cancer Family Study.

## Methods

### Study Population

The study was approved by the Institutional Review Boards at the University of Minnesota and Mayo Clinic and informed written consent was obtained from all participants. Details of the study design and methods have been published [28]. Briefly, breast cancer probands (n = 544) were ascertained at the Tumor Clinic of the University of Minnesota Hospital in 1944. A follow-up study was initiated in 1990 and telephone interviews detailed four-generation pedigrees of the probands and eligible sisters, daughters, nieces and granddaughters of the probands and women identified as the spouse of corresponding male first- and second-degree relatives of the probands. Interviews were attempted with 6,664 living female relatives 18 years of age (426 of the 544 families), and 6,194 (93% response) consented.

### Questionnaire Data

The telephone interview collected information on history of cancer, weight history, marital status, education, occupational class, medical history, reproductive history, oral contraceptive use, physical activity and history of smoking and alcohol intake. Menopausal status was determined from the response to a question of whether the participant had a menstrual period within the last year, excluding those brought on by hormones. Subjects were mailed a body measurement questionnaire that asked for replicated measures of current height and weight. Circumferences of the waist (2 inches above the umbilicus) and hips (maximal protrusion) were also obtained using a validated protocol [29].

### Subject Inclusion

The present study sample includes self-reported Caucasian women from 90 of 426 families with the most informative pedigrees chosen from simulation analysis, excluding the probands with breast cancer [28]. After the exclusion of 13 individuals due to consistent Mendelian errors across all markers, 889 individuals (756 women and 133 men) for whom we had blood-based DNA formed the basis of the genome screen. Males were excluded from analyses, although their genotypes were used in linkage analyses for determination of identity by descent (IBD) and to infer genotypes on female family members with missing DNA. The final sample consisted of 577 women who completed the telephone interview, returned the body measurement questionnaire and provided a DNA sample.

### Genotyping

DNA was genotyped for 403 microsatellite markers across the genome in the Mayo Clinic Cancer Center's Genotyping Shared Resource according to conditions suggested in the ABI Prism Linkage Mapping Set (PE Applied Biosystems, version 2.5, Foster City, CA). Genotypes were scored using an ABI 3100 or ABI 3730 DNA sequencer and ABI Genotyper v3.7 or ABI Genemapper v3.5 software. The average heterozygosity per marker was 0.77 and the average inter-marker distance was 8.9 cM. Of 361,021 called genotypes, 357,172 (98.9%) were Mendelian-consistent and used in analysis.

### Statistical analysis

To satisfy the normality assumptions required by variance components linkage analysis, we applied the t-rank transformation to the distributions of waist and hip circumferences and BMI; WHR was left untransformed. The relation between non-genetic covariates with known or suspected influence on obesity phenotypes [30] were examined separately by menopausal status using univariate regression and backward elimination regression models with retention *P *values < 0.10 (SAS Institute, Cary, NC); both approaches identified similar non-genetic covariates for the final models. We adjusted for age (continuous), parity (0, 1-2, or > 2 births), education (less than high school, some college, or college graduate), smoking (never, former smoker with < 20 pack years, former smoker with ≥ 20 pack years, current smoker with < 20 pack years, or current smokers with ≥ 20 pack years), physical activity (low, moderate, or high), and oophorectomy (no, one ovary or both removed). Since BMI is a measure of overall obesity, it was included in the models of waist or hip circumference and WHR to better isolate the genetic influences on body fat deposition in the waist and hip areas. BMI was defined in kg/m^2^. Because BMI was not normally distributed, we included the inverse (1/BMI) in continuous format in statistical models.

Multipoint IBD sharing probabilities were estimated in SIMWALK2 [31]. Heritability between the transformed and covariate-adjusted trait values was estimated with variance decomposition using maximum likelihood methods [32]. Univariate variance components linkage analysis was performed using the R library MULTIC [33,34] and a Fisher scoring algorithm to estimate genetic parameters while also adjusting for covariates. Genotype information at a locus was characterized by the probability that two related individuals share none, one, or two alleles IBD. Linkage was tested by a likelihood ratio test for the hypothesis that the QTL variance component is equal to zero compared with it being greater than zero [35]. The data were analyzed using no ascertainment correction because the phenotypes of interest did not lead to the ascertainment of the families. Univariate logarithm of odds (LOD) scores ≥ 2.0 were considered suggestive and LOD scores ≥ 3.3 were considered significant [36].

Multivariate variance component analyses have been shown to improve power to detect linkage over univariate procedures at loci with pleiotropic effects for genetically correlated phenotypes [37]. Evidence to support pleiotropy for obesity-related phenotypes in the current analysis includes the strong association at *LYPLAL1 *among women for both BMI and WHR [14]. We, therefore, performed bivariate linkage analyses using pair-wise combinations of obesity-related traits. Due to the greater degrees of freedom, higher LOD score thresholds are required to achieve comparable levels of statistical significance (e.g., ≥ 3.0 was considered suggestive and those ≥ 4.0 were considered significant) [36]. We performed multiple univariate and bivariate linkage analyses (these are multiple phenotypes and should not be confused with multiple testing). The LOD scores and corresponding *P *values serve chiefly to indicate the relative strength of evidence in favor of linkage. Similarly, when the bivariate LOD scores met the bivariate LOD score criteria for linkage, strength of the evidence in favor of pleiotropy was inferred if the bivariate *P *value was as small as or smaller than the component univariate *P *values.

## Results

Post-menopausal women were older, heavier and had greater waist circumference than pre-menopausal women, although no substantial difference was noted in hip circumference (Table 1). Among post-menopausal women, current users of hormones were, on average, younger (59.2 years, n = 138) than never (65.6 years, n = 193) or former (66.3 years, n = 74) hormone users. In age-adjusted analyses, current hormone users did not have significantly different WHR (*P *= 0.10), BMI (*P *= 0.13), and waist (*P *= 0.09) or hip (*P *= 0.25) circumferences than former or never hormone users (data not shown). Heritability of obesity traits was significantly greater than 0 (*P *< 0.001) for all estimates except waist circumference (*P *= 0.11) and WHR (*P *= 0.05) among pre-menopausal women (Table 2), suggesting most of the variation observed in these two phenotypes among pre-menopausal women is due to environmental and not genetic influences. Pre-menopausal women compared to post-menopausal women had higher heritability for BMI (*h*^2 ^= 94% versus *h*^2 ^= 39%, respectively) and for hip circumference (*h*^2 ^= 99% versus *h*^2 ^= 43%, respectively), and lower heritability for waist circumference (*h*^2 ^= 29% versus *h*^2 ^= 61%, respectively) and for WHR (*h*^2 ^= 39% versus *h*^2 ^= 57%, respectively). The high heritability of 99% for hip circumference among pre-menopausal women is likely an upper bound estimate. To verify this, we re-ran the hip circumference model among pre-menopausal women without any adjustment for covariates and observed *h*^2 ^= 83% (standard error = 0.11; data not shown). This indicates that hip circumference has a very strong polygenic heritability in pre-menopausal women.

**Table 1:** Descriptive characteristics among women in the Minnesota Family Study.

Variable^a^	Pre-menopausal women (n = 172)	Post-menopausal women (n = 405)
Age, years	42 (36-47)	63 (57-70)

BMI^b^, kg/m^2^	24.8 (21.9-29.3)	25.6 (23.1-29.1)

WHR^c^	0.79 (0.75-0.84)	0.85 (0.79-0.89)

Waist circumference, inches	31.0 (28.4-35.7)	34.0 (30.7-38.0)

Hip circumference, inches	39.7 (37.1-43.0)	40.0 (38.0-43.0)

^a^Median (25th and 75th percentiles).

^b^Body mass index.

^c^Waist to hip ratio.

**Table 2:** Heritability estimates^a ^for obesity-related traits among women in the Minnesota Family Study.

Obesity trait^b^	Pre-menopausal women (n = 172)	Post-menopausal women (n = 405)
BMI	0.94	0.39

WHR	0.38	0.57

Waist circumference	0.29	0.61

Hip circumference	0.99	0.43

^a^Heritability is the proportion of interindividual phenotypic variance explained by additive genetic factors, measured on a scale from 0 to 1. Heritability estimates were significantly greater than 0 (*P *< 0.001) except waist circumference (*P *= 0.11) and WHR (*P *= 0.05) among pre-menopausal women. Obesity traits were adjusted for age, parity, education, smoking, physical activity, and oophorectomy. BMI was not adjusted in model estimates of heritability.

^b^BMI, body mass index; WHR, waist to hip ratio.

**Figure 1:**
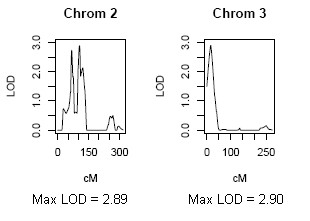
**Maximum LOD scores achieved for univariate linkage to the BMI trait among pre-menopausal women**.

**Figure 2:**
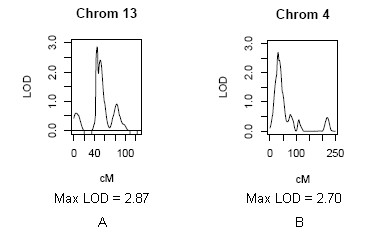
**Maximum LOD scores achieved for univariate linkage to the hip circumference trait among pre-menopausal women (A) and post-menopausal women (B)**.

For the BMI phenotype, the highest level of evidence for genetic linkage was achieved on chromosomes 2p21-p22 and 3p26 among pre-menopausal women, based on LOD scores satisfying suggestive evidence (e.g., LOD = 2.89 and 2.90, respectively) (Table 3 and Figure 1). For the hip circumference trait, the highest level of evidence for genetic linkage was achieved on chromosome 13q13 among pre-menopausal women (LOD = 2.87) and on chromosome 4p15.3 among post-menopausal women (LOD = 2.70) (Table 3 and Figure 2). The WHR trait also showed suggestive evidence for linkage among pre-menopausal women on chromosome 1q21 (Table 3).

**Table 3:** Univariate multivariable-adjusted^a ^multipoint linkage (LOD ≥ 2.0) at chromosomal regions with obesity-related traits.

Sample	Position	Trait^b^	Peak LOD score	Marker closest to peak LOD score	Genetic map distance of marker (cM)^c^	Physical map position of marker (bp)^d^	Marker interval of marker closest to peak LOD score	Physical map position of marker interval (bp)^d^	Nearest gene on genome to marker	Physical map position of nearest gene on genome (bp)	Nearest gene previously reported with trait	Physical position of nearest gene previously reported with trait (bp)^d^
Pre-menopausal	1q21	WHR	2.09	D1S498	143.75	149,568,120	D1S252-D1S484	117,358,294-159,034,332	*PI4KB*	149,531,037	*LYPLAL1 *^*e*^	217,413,846
	
											*RASAL2 *^*e*^	176,330,253
	
												
	
	2p21-p22	BMI	2.89	D2S2259	68.78	42,850,051	D2S367-D2S391	34,294,652-46,265,269	*HAAO*	42,847,734	*TMEM18 *^*e*^	657,975
	
												
	
	3p26	BMI	2.90	D3S1297	14.83	2,013,403	D3S1297-D3S1304	2,013,403-6,894,583	*CNTN4*	2,117,247	*GHRL *^*f *^ *PPARG *^*f*^	10,302,434 12,304,349
	
	3p26	BMI	2.90	D3S1304	14.83	6,894,242	D3S1297-D3S1263	2,013,403-11,492,535	*GRM7*	6,877,927	*GHRL *^*f *^ *PPARG *^*f*^	10,302,434 12,304,349
	
												
	
	13q13	HC	2.87	D13S218	34.15	37,930,231	D3S2338-D3S1266	16,824,399-27,932,698	*UFM1*	37,822,018	*HTR2A *^*f*^	46,305,514
	
												
	
	13q14	BMI	2.60	D13S263	44.42	40,978,920	D13S218-D13S153	37,930,231-47,789,009	*C13orf15 (RGC32)*	40,929,712	*HTR2A *^*f*^	46,305,514
	
	13q14	BMI	2.60	D13S153	44.42	47,788,735	D13S263-D13S156	40,978,920-73,555,776	*RB1*	47,775,884	*HTR2A *^*f*^	46,305,514
	
												

Post-menopausal	4p15.3	HC	2.70	D4S403	25.0	13,359,926	D4S2935-D4S419	6,611,782-18,458,207	*BOD1L*	13,179,464	*GNPDA2 *^*e*^	44,398,926
	
												
	
	5q21	HC	2.01	D5S433	115.1	103,990,522	D5S644-D5S2027	95,838,450-111,173,613	None ^g^		*CARTPT *^*f *^*NR3C1 *^*f *^*ADRB2 *^*f*^	71,050,750 142,637,689 148,186,349
	
	5q22	HC	2.01	D5S2027	115.1	111,173,318	D5S433-D5S471	103,990,522-119,077,177	*STARD4*	110,861,921	*CARTPT *^*f *^*NR3C1 *^*f *^*ADRB2 *^*f*^	71,050,750 142,637,689 148,186,349
	
	14q13	HC	2.51	D14S70	34.15	33,528,945	D14S275-D14S288	25,766,613-43,171,795	*EGLN3*	33,463,174	*ESR2 *^*f*^	30,234,561

^a^Adjusted for age, parity, education, smoking, physical activity, and oophorectomy. BMI was included in the models of waist circumference and WHR, and of hip circumference and WHR.

^b^BMI, body mass index; WHR, waist to hip ratio; HC, hip circumference.

^c^Genetic map distance in centimorgans (cM) is the position of genetic markers relative to each other along a chromosome in terms of recombination frequency, rather than as specific physical distance. One cM represents approximately 1% recombination frequency. The greater the recombination frequency between two genetic markers along a chromosome, the farther apart physically they are assumed to be.

^d^HapMap Genome Browser, NCBI Build 36.

^e^From genome-wide association studies.

^f^From linkage studies.

^g^None within 500 kb.

**Figure 3:**
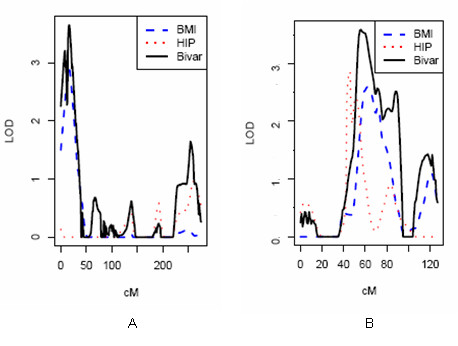
**Maximum LOD scores achieved for bivariate linkage to the BMI and hip circumference traits among pre-menopausal women at chromosome 3p26 (A) and chromosome 13q13-q14 (B)**.

The bivariate linkage analyses provided suggestive evidence (LOD ≥ 3.0) for 12 loci with pleiotropic effects on pairwise combinations of obesity traits (Table 4). The strongest evidence was among pre-menopausal women on chromosomes 3 and 13, where the bivariate LOD scores for the combination of BMI and hip circumference traits were 3.65 at 3p26 and 3.59 at 13q13-q14 (Figure 3A and 3B). The bivariate genetic correlations between these two traits were 0.85 at 3p26 and 0.53 at 13q13-q14 (data not shown). Among post-menopausal women, there was no evidence of loci with pleiotropic effects as indicated by LOD scores ≥ 3.00 on pairwise combinations of obesity traits.

**Table 4:** Bivariate multivariable-adjusted^a ^multipoint linkage (LOD ≥ 3.0) at chromosomal regions with obesity-related traits among pre-menopausal women.

Position	Trait^b^	Peak LOD score	Marker	Peak LOD position (cM)
2p23	BMI-HC	3.30	D2S165	47.90

2p23	BMI-WHR	3.17	D2S165	48.47

				

3p26	BMI-HC	3.65	D3S1304	14.05

3p26	BMI-WC	3.00	D3S1304	14.83

				

11q22	BMI-HC	3.14	D11S898	104.45

				

13q13	BMI-HC	3.59	D13S218	40.88

13q13	HC-WHR	3.01	D13S218	41.47

				

13q14	BMI-HC	3.59	D13S263	40.88

13q14	HC-WHR	3.01	D13S263	41.47

13q14	WC-WHR	3.00	D13S263	42.66

13q14	BMI-WHR	3.28	D13S263	43.25

^a^Adjusted for age, parity, education, smoking, physical activity, and oophorectomy. BMI was included in the models of waist circumference and WHR, and of hip circumference and WHR

^b^BMI, body mass index; WHR, waist to hip ratio; WC, waist circumference; HC, hip circumference.

## Discussion

Over 250 QTL for human obesity-related phenotypes have been published from over 61 genome-wide scans [6] and from over 10 GWAS http://www.genome.gov/GWAStudies. Only a minority of these, however, examined WHR, waist circumference or hip circumference phenotypes [9,14,38] and, to our knowledge, none published on the genetic regulation of fat deposition in women by menopausal status.

Early observations reported increased visceral fat in post-menopausal compared to pre-menopausal women independent of age using objective assessments of body composition such as CT scans [23]. Samaras and colleagues [39] measured total and central fat using dual-energy X-ray absorptiometry (DEXA) among 216 post-menopausal twin pairs. They reported heritability estimates of 56% for total fat and 64% for central fat that did not appear to be explained by the same genetic factors, suggesting the two traits are regulated independently. Pre-menopausal women were not studied. We report similar heritability estimates among post-menopausal women in our study for waist circumference (*h*^2 ^= 61%) and provide additional evidence that heritability for this trait is lower, and not statistically significant, among pre-menopausal women (*h*^2 ^= 29%). These observations support two conclusions. First, low heritability for waist circumference (and for WHR) among pre-menopausal women suggests most of the phenotypic variance is due to environmental influences. Second, the higher heritability for these two phenotypes among post-menopausal women suggests additive genetic factors explain a significantly larger proportion of the inter-individual variation. If true, then interventions that modify environmental contributions to abdominal fat distribution (e.g., increased physical activity, decreased total energy consumption) should target women in their pre-menopausal years, when such interventions may be more effective.

The heritability for hip circumference among pre-menopausal women (*h*^2 ^= 99%) is much larger than among post-menopausal women (*h*^2 ^= 43%). Along with higher heritability for hip circumference among pre-menopausal women, we also found suggestive evidence for linkage. We showed that the phenotypes of BMI and hip circumference were linked to the same loci at chromosome 2p21-p22, chromosome 3p26, and chromosome 13q13-q14. The high genetic correlation between these traits and their linkage to the same chromosomal regions at 3p26 and 13q13-q14 provides evidence for pleiotropic effects of these loci among pre-menopausal women. Because BMI is a ratio measure of weight and height, we cannot rule out that some of the linkage at regions 2p22-2p23 and 13q14 is with the height phenotype, as reported by several GWAS of height http://www.genome.gov/GWAStudies. In contrast, there was no evidence for pleiotropy among post-menopausal women; however, the hip circumference trait showed evidence of linkage to chromosome 4p15.3 and chromosome 14q13 indicating the influence of independent loci. Although we found higher heritability for waist circumference among post-menopausal women compared to pre-menopausal women, we did not find evidence for linkage with waist circumference at loci in either of the two groups of women. Reasons for this might include allelic effects that are smaller or causal alleles that are too rare to detect with the given sample size [40], or confounding by unaccounted environmental influences. For instance, if the resemblance of first-degree family members is partly due to common environmental effects, then an estimate of heritability that is based on their resemblance will be biased upwards [41]. However, our heritability estimate for waist circumference among post-menopausal women was similar to that reported by Samaras and colleagues [39], and the circumferences in this analysis were based on actual measurements that have been shown to be obtained reliably and with good precision [29].

To our knowledge, only two GWAS reported obesity phenotype associations separately by gender. Lindgren and colleagues [14] reported genome-wide significant associations with WHR near *LYPLAL1 *at 1q41 among women before adjusting for BMI (P = 2.6 × 10^-8^), which attenuated following BMI adjustment (P = 4.3 × 10^-6^), whereas the association among males was not statistically significant (P = 0.50). Associations were also seen for BMI (P = 1.9 × 10^-4^) and waist circumference (P = 0.01) at these loci among women but not for men [14]. The second GWAS reported associations with waist circumference for *NRXN3 *at 14q31 among women (P = 0.0005) and men (P = 0.001), which was no longer statistically significant following BMI adjustment [38]. These studies provide evidence that genetic determinants of adiposity vary by sex, and support further investigation of the GWAS candidates by menopausal status.

Genes previously-reported to be associated with obesity and that reside within marker intervals at our peak LOD scores include *GHRL, PPARG, HTR2A *and *ESR2 *(Table 3). They influence appetite and the pathology of numerous diseases including obesity [24,42-46]. Sowers and colleagues [24,42] reported findings that suggest changes in ghrelin concentrations in the peri-menopause may precede increases in waist circumference. Ghrelin can stimulate feeding and body weight gain by neuroendocrine mechanisms [47,48]. In humans, PPARG activation causes a shift in fat distribution from visceral to subcutaneous fat, increases plasma adiponectin, decreases plasma resistin and suppresses macrophage production of inflammatory markers - all which result in improved insulin sensitivity and glycemic control [43]. Treatment with estrogen prevented intra-abdominal fat deposition [26,27] and promoted fat oxidation in muscle through up-regulation of PPARG expression [49]. If the absence of estrogen leads to abdominal fat deposition, it is possible that reduced PPARG expression may act cooperatively to potentiate metabolic syndrome-related symptoms that are often associated with increased waist girth [44]. *HTR2A *encodes a serotonin receptor and serotonin is a key mediator in the control of appetite, weight regulation and body weight distribution [50] (also reviewed in [45]). Low cerebrospinal fluid levels of serotonin metabolites have been found in women with primarily abdominal obesity [51]. Furthermore, serotonin responsivity declines after menopause [52], and estrogen treatment in post-menopausal women both decreases brain 5-HT2A receptors [53] and prevents abdominal fat deposition [26,27], possibly by restoring the sensitivity of the receptors to circulating serotonin. Interestingly, estrogen receptor beta (ERβ) is the receptor responsible for serotonergic neurotransmission in primates [46]. *ESR2*, which encodes ERβ, is within the marker interval at 14q23 that linked to hip circumference among post-menopausal women in our study. In addition, our linkage analysis identified novel regions and putative genes that may also be associated with obesity-related phenotypes including *PI4KB*, *HAAO*, *CNTN4*, *GRM7*, *UFM1*, *RB1*, *BOD1L*, *STARD4*, *EGLN3 *and *NRN1*. Interestingly, some of these genes appear to play a role in neurological disorders [54], or neuronal development or signaling [55-57], similar to those genes identified recently in the GWAS [12,38].

Although our findings point to plausible genetic influences for obesity traits by menopausal status, this study also has potential limitations. The findings were based on a small sample size with a relatively small number of genetic markers. We reduced the possibility that evidence for linkage was due to a few families with extreme phenotypes by ensuring the phenotypes were normally distributed. Further, the genetic effect on phenotypes such as BMI may decrease with age as one loses height or muscle mass [39]. Ideally, serial measurements would strengthen and serve to validate genetic analyses of quantitative obesity-related phenotypes [58].

## Conclusions

In summary, these results provide a genetic basis for fat deposition that differs by menopausal status, and suggests the same loci encode genes that influence general obesity (BMI) and specifically hip circumference among pre-menopausal women. However, lower heritability among pre-menopausal women for waist circumference and WHR suggests that pre-menopausal waist girth may be influenced to a greater extent by controllable environmental factors than post-menopausal waist girth. Although requiring confirmation, it is possible that targeted interventions among pre-menopausal women may prevent or attenuate post-menopausal abdominal weight deposition. Future studies will need to disentangle the precise mechanism(s) between the loci/genes reported in this investigation with the environment to aid in our understanding of obesity phenotypes among pre-menopausal and post-menopausal women for chronic disease prevention.

## Competing interests

The authors declare that they have no competing interests.

## Authors' contributions

LEK contributed to analysis and interpretation of data, and drafted and revised the manuscript; CMV, TAS and FJC conceived the study design, acquired the data and funding, and contributed to manuscript revisions; EJA, MdA, VSP and AW implemented and interpreted the statistical analysis and contributed to manuscript revisions; JMC and CAH performed and interpreted the genotyping data; all authors read and approved the final manuscript.

## Pre-publication history

The pre-publication history for this paper can be accessed here:

http://www.biomedcentral.com/1471-2350/11/156/prepub
